# Commentary: High-intensity Intermittent Training vs. Moderate-intensity Intermittent Training: Is It a Matter of Intensity or Intermittent Efforts?

**DOI:** 10.3389/fphys.2017.00370

**Published:** 2017-05-30

**Authors:** Paulo Gentil, Fabrício B. Del Vecchio

**Affiliations:** ^1^Faculdade de Educação Física e Dança, Universidade Federal de GoiásGoiânia, Brazil; ^2^Escola Superior de Educação Física, Universidade Federal de PelotasPelotas, Brazil

**Keywords:** high intensity interval exercise, high intensity intermittent training, physical fitness, high intensity exercise, cardiorespiratory fitness

We read with great interest the article by Jimenéz-Pavón and Lavie (2017). While we agree with the value of prescribing and studying moderate-intensity interval training (MIIT), there are important aspects that need to be clarified. The authors raise the point that “intensity” is not the only difference between high-intensity interval training (HIIT) and aerobic continuous training (ACT). The authors cite three studies to support the notion that the intermittent nature of the exercise and not the intensity may be responsible for the results. However, the studies cited do not seem to support the points raised. Rakobowchuk et al. (2012) trained two groups at the same intensity (120% of the peak work rate obtained in a ramp-incremental test), but with different working parameters (repeated intervals of 10:20 s vs. intervals of 30:60 s); therefore, this cannot be considered a comparison between HIIT and MIIT. In the study conducted by Alkahtani et al. (2013), the protocols differed in intensity but also in interval duration (5 min vs. 30 s). Therefore, one cannot use the study to make inferences about training intensity as the other parameters were not equal. Moreover, in the study conducted by Alkahtani et al. (2013), the group that exercised at higher intensity performed 30 s at 90% of the intensity equivalent to VO2peak, interspersed with 30 s of passive rest. Although this intensity was higher than that performed by the other group (20% above 45% VO2peak), it is still lower than reported in previous studies using the same interval duration (Billat et al., 1999, 2000a,b; Billat, 2001a,b; Racil et al., 2013, 2016).

The only study that has really compared different intensities is Racil et al. (2013). The authors equated the number of bouts, rest intervals, etc. In this study, the decreases in waist circumference, triglyceride and total cholesterol were significant only in the HIIT group. In addition, increases in maximal aerobic speed and decreases in percentage of body fat, low-density lipoprotein cholesterol (LDL-C), and insulin were higher in the HIIT group than in the MIIT group. Although the increases in VO2peak were significant for both groups (7.7% for HIIT and 5.2 for MIIT), only the HIIT group showed a significant increase compared to the control group. Therefore, the only study that made comparisons with different intensities while keeping the other parameters constant clearly favored HIIT exercises. While this may not answer the question of whether intensity or the intermittent nature of interval training is the most important parameter, it suggests that HIIT promotes better results than MIIT.

We must recall, however, that defining HIIT intensity is not a matter of “the more the better.” The efficiency of HIIT seems to be a matter of choosing the adequate intensity. In this regard, Raleigh et al. (2016) investigated the effects of HIIT intensity on training-induced adaptations in VO2peak and VO2 kinetics. The authors compared the effects of HIIT (1 min of effort per 1 min of rest) targeting 80, 115, or 150% of the intensity equivalent to VO2max while matching total work performed. According to the results, increases in VO2peak were greater in the group that trained at 115% than in the group that trained at 80%. No differences were observed between the groups that trained at 150 and 80% as well as between the groups that trained at 150 and 115% of iVO2max. The greatest proportion of non-responders was observed in the group that trained at lower intensity and the greatest proportion of responders was found in the group that trained at 115%. Therefore, one should not advocate for or against high intensity, but rather for adequate intensity.

Jimenéz-Pavón and Lavie (2017) suggested that high-intensity exercise can sometimes deter physically inactive and unfit people; however, this has not been found in previous studies. Indeed, Guiraud et al. (2011) reported that patients with chronic heart disease preferred HIIT to ACT. Jung et al. (2014) reported that adults with prediabetes can adhere to HIIT at a level that is greater than ACT. Furthermore, Jung et al. (2015) compared HIIT (1 min~100% W peak and 1 min~20% W peak for 20 min), ACT at moderate intensity (~40% W peak for 40 min) and ACT at high intensity (~80% W peak for 20 min). According to the results, participants reported greater enjoyment related to HIIT compared to the other protocols and 62% of the participants reported a preference for engaging in HIIT.

Although we agree that enjoyment ratings might be reduced when HIIT is strenuous, chronic training may lead to increased enjoyment due to an increase in achievement. In this regard, Heisz et al. (2016) randomly assigned sedentary young adults to HIIT (1 min~90–95% peak HR followed by 1 min at 30% PPO for a total of 20 min) or ACT (27.5 min~70–75% peak HR) for 6 weeks. Enjoyment of HIIT increased with training, whereas enjoyment of ACT remained constant but lower.

While we agree that MIIT might be an interesting strategy at some points and that more studies regarding the topic are needed, the references presented and the limitations raised by the authors do not seem to support the points raised.

## Author contributions

PG and FD conceived, drafted, and revised the manuscript. All authors read and approved the final manuscript.

### Conflict of interest statement

The authors declare that the research was conducted in the absence of any commercial or financial relationships that could be construed as a potential conflict of interest.
